# Automated parasitological diagnosis in clinical microbiology laboratories

**DOI:** 10.1038/s41598-021-92441-3

**Published:** 2021-06-23

**Authors:** Gema Fernández-Rivas, Belén Rivaya, Nona Romaní, Jun Hao Wang Wang, Mireya Alcaide, Lurdes Matas

**Affiliations:** 1 Microbiology Department, Clinical Laboratory North Metropolitan Area, University Hospital Germans Trias I Pujol, Badalona, Spain; 2 Department of Genetics and Microbiology. Autonomous University of Barcelona, Barcelona, Spain; 3 Present Address: CIBER in Epidemiology and Public Health (CIBERESP), Madrid, Spain

**Keywords:** Infectious diseases, Microbiology, Clinical microbiology, Infectious-disease diagnostics, Parasitology

## Abstract

Although there is a low prevalence of parasitological infections in Europe, the diagnosis of intestinal parasites is still difficult and laborious for microbiology laboratories. Currently, antigen detection assays and molecular biology allow a more accurate diagnosis, but these techniques have limitations as they cannot detect all the possible parasites present in the samples. The objective of the study was to evaluate the accuracy and the usefulness of automated microscopy SediMAX2 (77 Elektronika, Budapest, Hungary) in the detection of parasitic infections from feces. A total of 197 formol-fixed stool samples were processed in parallel by wet mount examination and by SediMAX2. Sensitivities, specificities and predictive values were analyzed, reaching a sensitivity of 89.51% and a specificity of 98.15% and a very good positive predictive value (99.22%). SediMAX2 is a good tool for a reliable diagnosis of intestinal parasitic infections. The rapid processing and the flexibilty of storage of images analyzed make its incorporation into the day to day laboratory routine recommendable.

## Introduction

Parasitogical diagnosis needs to be improved. Part of the effort must be focused on recording clinical symptoms, travel history and geographic location of the patient, because this information will help to define the suspected etiology. But another important aspect is that the parasitological techniques employed in most of the clinical microbiology laboratories in Europe still rely on microscopy and are observer dependent. The low prevalence of parasitic infections in Europe makes the diagnosis of intestinal parasites more difficult and laborious for microbiology laboratories. In recent years, there has been a tremendous effort to focus research on the development of new diagnostic methods, such as serological, molecular, and proteomic approaches^1^, but these techniques also have limitations due to the fact that they do not detect all possible parasites; also they are expensive and are not available for all clinical microbiology laboratories. Molecular assays have emerged as the solution for diagnosis of a lot of infectious diseases and several targets have been used for the diagnosis of parasitic infections but, many of them are locally designed solutions and they are not standardized^2^.

Several in-house methods have been developed for different parasitic infectious^3,4^, but is in diagnosis malaria that this new technology has triggered the development of new and available diagnostic kits, which yield much better results^5^ . Specially remarkable is the development of loop-mediated isothermal amplification (LAMP)^6^, which has attracted a lot of attention in the field of parasitology. The future of malaria diagnosis has also changed with the incorporation of a new microscopic platform which incorporates Parasight, which is an enhanced computer vision device for the diagnosis of malaria^7^ which is able to provide highly sensitive, faster and more accurate malaria evaluations than the microscope malaria diagnosis. This is a big step in the evolution of classical microscopic diagnostics, which can be applied to other areas of parasitology.

In 2015, a group of Italian researchers published an interesting article in which they evaluated the accuracy of an autoanalyzer, which is used for the diagnosis of urinary infections, the sediMAX 1 (77 Elektronika, Budapest, Hungary)^8^, and additionally, they evaluated an improved version for the detection of protozoans^9^.

Digital microscopy is already being used in pathology departments with the advent of Whole-Slide Imaging, and in this aspect, SediMAX 2 could be the first step for virtual microscopy in parasitology.

We aimed to evaluate the accuracy and the usefulness of the SediMAX2 for the diagnosis of intestinal parasitic infections from formalin-fixed stools and its effectiveness in a high throughput laboratory.

## Material and methods

### Study design

This was a cross-sectional observational study designed for the clinical evaluation of SediMAX2 compared with microscopy for the routine diagnosis of gastrointestinal parasitic infections at the Microbiology Department of the Germans Trias i Pujol University Hospital (HUGTiP, Badalona, Spain).

### Study population and clinical samples

**Figure 1:**
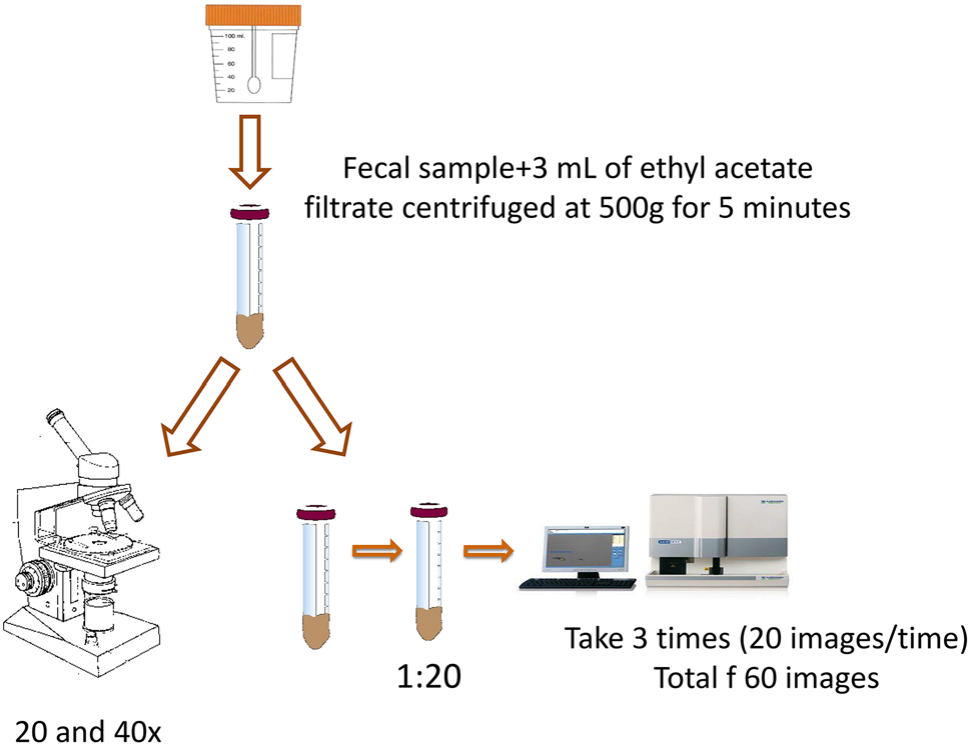
Flow chart of the total process.

During the period January 2016 to December 2017 a total of 197 preselected fecal samples from 178 patients with suspected intestinal parasitic infection that had been fixed with sodium acetate-acetic acid-formalin (SAF) (Universal System 50 ml SAF, Durviz, Valencia, Spain) and processed in the Microbiology Department of the HUGTiP were included in the current study. Stools were received from Monday to Friday during the morning schedule and processed at our hospital clinical microbiology laboratory. These samples came from patients with suspected parasitic infections from two different populations: (i) patients at the emergency department or hospitalized at the HUGTiP and (ii) patients in primary care settings. Positive and negative samples were selected after microscopic examination and at the same, were analyzed by SediMAX2 (Fig. 1).

### Stool sample processing

Before processing, the samples were examined microscopically for ova and parasites. First, fixed stools were diluted with 3 ml of ethyl acetate and then they were filtrated by centrifugation at 500*g* for 5 min. Sediment was observed at 20 and 40 × by optical microscopy in order to identify parasite structures.

Subsequently, the samples were analyzed using the automatic microscopy sediment analyzer, SediMAX2. After concentration of the fixed feces, the sediment was subsequently diluted with saline solution (1:20) and was analyzed by SediMAX2 as described by Intra et al.^8^. The autoanalyzer homogenizes and transfers 20 µl of the diluted sample into special disposable cuvettes, which are centrifuged for a few seconds. SediMAX2 whole-field high definition images were obtained. All images were stored on the computer to be reviewed by a second independent reader. For each sample, a triple analysis was carried out with SediMAX2, so a total of 60 images were reviewed. Each time the SediMAX2 takes the sample, a total of 20 images are obtained and stored. The software of the SediMAX2 was customized in order to take 3 three times the same sample to have the 60 images. The image and the SediMax2 process was performed by another and independent technician.

### Statistical analysis

The agreement among two methods was evaluated using the Kappa coefficient (κ; CI 95%). Kappa result be interpreted as follows: values ≤ 0 as indicating no agreement and 0.01–0.20 as none to slight, 0.21–0.40 as fair, 0.41–0.60 as moderate, 0.61–0.80 as substantial, and 0.81–1.00 as almost perfect agreement. Sensitivity (Se), specificity (Sp), positive and negative predictive values (PPV and NPV) for SediMAX2 were also calculated using OpenEpi software, www.openepi.com (Emory University. Atlanta. USA).

### Ethics statement

Ethical approval from the Ethics Committee Research of Germans Trias i Pujol University Hospital Ethics Committee was obtained (PI-17–232) and the need for informed consent was waived. All methods were performed in accordance with the relevant guidelines and regulations.

### Ethics approval and consent to participate

The study was approved by the Ethics Committee at our institution (PI-17-232). The need for informed consent was waived.

### Consent for publication

The authors consent this paper for its publication.

## Results

Out of the 197 samples processed from 178 patients, 146 were positive; 124 presented single infection and 22 co-harbored 2 or 3 parasites and 54 were negative by wet mount examination. Regarding the positive samples with a single infection: 78 were *G. lamblia*, 10 *B. hominis*, 9 *Entamoeba coli*, 1 *Dientamoeba fragilis*, 3 *Entamoeba hystolitica/dispar*, 3 *Endolimax nana*, 5 *Enterobius vermicularis*, 3 *Hymenolepis nana*, 3 *Iodamoeba bustchlii*, 2 *Strongyloides stercoralis*, *4 Ascaris lumbricoides*, 2 *Trichuris trichura* and 1 *Taenia* spp. From the mixed infection samples, 2 were positive for *H. nana* and *G. lamblia*; 2 for *E. nana* and *B. hominis*; 2 for *E. coli* and *E. nana,* 1 for *G. lamblia*, *E. nana* and *B. hominis*; 1 for *G. lamblia*, *E. nana*, *E. coli* and *B. hominis*; 3 for *E. nana*, E*. coli* and *B. hominis*; 4 for *E. coli* and *B. hominis*; 1 for *E. hystolitica*, *Entamoeba hartmanii* and *E. nana* and 1 for *G. lamblia*, *E. hystolitica* and *B. hominis*; 1 for *G. lamblia* and *E. hystolitica* and 1 for *A. lumbricoides* and *E. nana*. Values of SE, SP, PPV, NVP and *Kappa* index were (IC 95%): 89.51%; 98.15%, 99.22%, 77.94% and 0.81 respectively compared with the wet mount examination.

Out of 197 samples studied there were 16 discrepancies shown in Table 1.

**Table 1:** Discordant results between SediMAX2® and wet mount examination.

Patient number	Wet mount examination	SediMAX2®
1	*E.coli*	Negative
2	*G.lamblia*	Negative
3	*G.lamblia*	Negative + *B.hominis*
4	*G.lamblia*	Negative
5	*E.vermicularis*	Negative
6	*I.bustchlii*	Negative
7	*G.lamblia*	Negative
8	*E.vermicularis*	Negative
9	*S.stercoralis*	Negative
10	*A.lumbricoides*	Negative
11	*A.lumbricoides*	Negative
12	*E.vermicularis*	Negative
13	Negative	*B.hominis*
14	*G.lamblia*	Negative
15	*G.lamblia*	Negative
16	Negative	*G. lamblia*

Focusing in the discrepancies, there are 2 samples in which detected parasites have debatable clinical significance such as *E. coli* or *I. bustchlii* and 1 sample with a positive result for *B. hominis* by SediMAX2. Considering these results as negative, the data obtained for SE, SP and predictive values were recalculated and the accuracy improved with SE, SP, PPV, NVP and *Kappa* index (95% IC) of 90.78%; 100%; 100%; 81.16% and 0.8484 respectively. Three samples with *E. vermicularis* were included although the definitive diagnosis should be performed with the "Scotch test", cellulose-tape slide test on the perianal skin.

**Figure 2:**
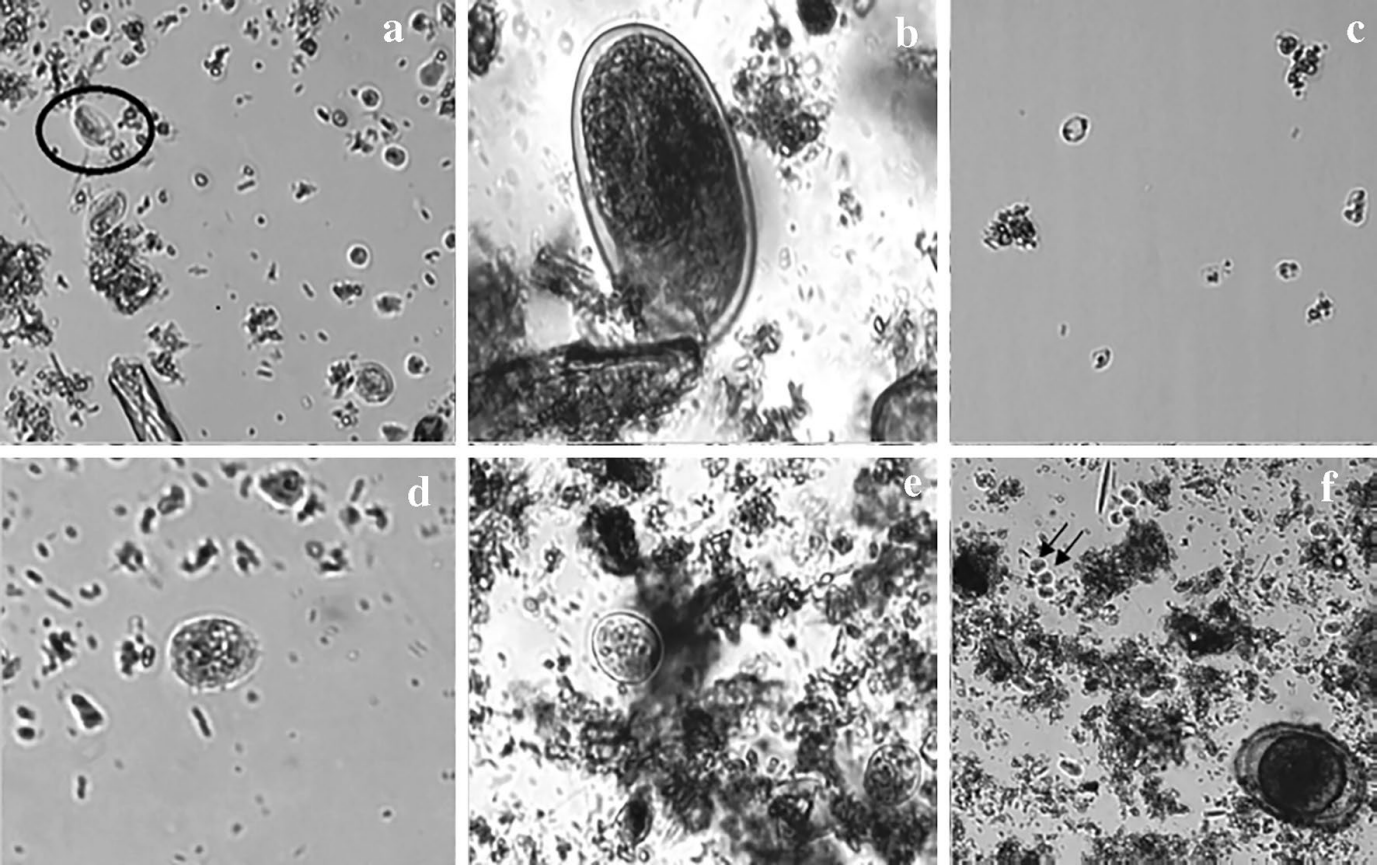
Images from SediMAX2 software. A. *Giardia lamblia* cysts (circled). B. Hookworm egg. C. *Blastocystis hominis* vacuolar stage. D. *Entamoeba hystolitica/dispar* cyst. E. *Entamoeba coli* cysts. F. Double infection of *Endolimax nana* cysts (arrows) and *Ascaris lumbricoides* egg.

In all samples 60 images were processed and reviewed by an independent reader, but in 101 of the 143 positive samples (Fig. 2), the detection of the parasite was performed with only the first 20 images, reducing one of the problems of parasitological diagnosis by making it less time consuming. In 18 cases 40 images were needed and in 23 cases all 60 images were reviewed for a correct diagnosis. Additionally, a squared 15 × 15 µm was installed to allow the measurement of the structures for a correct parasitological evaluation. Despite the time savings in gaining these results, the evaluation of all images is recommended.

## Discussion

It is of great importance for microbiology departments to be able to correctly diagnose parasitic infections in a reliable and cost-effective way to in order to avoid further disease transmission and chronic illnesses. However, currently microscopic examination of stool samples for the detection of cysts, trophozoites and ova remains the diagnostic method of choice for many laboratories. This method requires technical expertise and is laborious and time-consuming. Additionally, it lacks sensitivity if there are low levels of infection^10^. It should also be noted that for many years, the technology for the diagnosis of parasitic infections has been neglected in terms of laboratory development.

The limitations of microscopy and antigen detection tests have encouraged parasitologists to move towards the use of genomic amplification methods made possible with the advent of molecular diagnostics, but they still remain underused^2,11^. Step by step, antigen detection tests for *G. lamblia*, *Cryptosporidium* spp and *Entamoeba hystolitica* have been widely introduced into the day-to-day laboratory workflow, especially in those laboratories with lower capacities for parasitological diagnosis. There are even several tests cleared by the FDA and they are associated with a significant improvement^10^.

Despite the growth in international travel and migration from endemic areas, in our settings *G. lamblia, Cryptosporidium* spp and *E. vermicularis* are still the most prevalent pathogens. In high throughput laboratories, tools for detection of these parasites are essential. The results obtained for *G. lamblia* diagnosis suggest that SediMAX2 could be a good tool and it can be implemented for the detection of this protozoan (SE, SP, PPV, NPV and Kappa index; 89,29%; 98,15%; 98,68%; 85,48% and 0.85 (0.68–1.017)respectively for *G. lamblia*). Of the 84 positive samples for *G. lamblia*, in 9 cases SediMAX2 was not able to detect them with a total agreement of 92.75 (including negative samples). However, it is important to remember that a negative result does not rule out parasitic infection because parasites (particularly *G. lamblia*) have an intermittent shedding^12^ and the probability of parasite detection increases more than 95% when 3 stools are tested^13^, so serial parasitological studies are still needed to confirm a high suspicion of *G. lamblia* infection in case of a previous negative result. Additionally we think that in clinical microbiology laboratories, microscopic independent techniques should be improved in order to enlarge the number of *G. lamblia* infections. In this aspect, this is a limitation in this study because we have only evaluated one sample. For other protozoans SediMAX2 was able to detect the pathogenic protozoans, but only 4 *E. hystolitica/dispar* positive samples were included in the study.

In case of worm infections, all eggs were properly identified with the exception of *E. vermicularis* (in all three cases), 2 *A. lumbricoides* and 1 case of *S. stercoralis* larvae. In the other 15 worm infections from 13 patients, eggs were detected in all cases. The discrepancies in the worm infections must be explained by the fact that in case of *E. vermicularis*, a stool wet mount examination is not the recommended method of diagnosis and even when its presence is detected in the microscopic mount, a tape slide-test must be sent to the laboratory for a correct diagnosis. For *S. stercoralis*, it is known that the visualization of rhabditiform larvae in stools is not always possible and it has to be complemented with other techniques such as a serology-based test. Diagnosis of *S. stercoralis*, is often delayed due the presence of subclinical or poorly-symptomatic cases and the usual low parasite load and irregular larvae output. These characteristics mean that this worm is also known as “the worm that there, but is unseen”^14^.

The most important aspect of this automatic microscopic system is that it considerably reduces hands-on time. With a huge capacity for the storage of images, this system could also reduce the time on the microscope while maintaining a high positive predictive rate, without reducing the quality of the diagnoses. This could be a good option to make flexible the parasitologist’s work.

Parasitological diagnosis is very labor-intensive and relies exclusively on the experience of trained technicians. Thus, it is difficult to maintain enough people with expertise in diagnostic medical parasitology on the staff of a laboratory. A recognized image system based in the same principle as that used for the analysis of urines should be developed by biomedical engineering to provide new tools to detect cases of intestinal parasitic infections. Additionally, improvements in the sample preparation processes to avoid the inclusion of detritus, which can hinder the interpretation of images would help to improve parasitological diagnose even more.

This technology is now being developed by a Malaysian group, who have already obtained good results for *A. lumbricoides* and *Trichuris trichura* eggs, but there is still a lack of options for detecting other helminthes and trophozoites. Another advantage of the Malaysian group’s software is that it is able to count the number of parasites which have been detected for each single patient and it also provides a user-friendly interpretation. This mean it displays results while reducing manpower needed and also increasing the speed, as it reaches speeds of 1–2 s/image, faster than conventional methods^15^.

This study in an initial trial to implement engineering with medical practice in order to make diagnosis of intestinal parasites easier for microbiologists. There are still opportunities for improvement, especially in high throughput laboratories, where diagnosis is almost exclusively manual. In those cases, a two-step algorithm including antigen detection and digital microscopy could be useful to help parasitologists in their day-to-day workload. This algorithm could be based on microscope independent tools such as antigen or molecular techniques^16^ as the first step to detect the more prevalent parasites (*Cryptosporidium* spp and *G. lamblia*) followed by other microscope based tool in cases in whom other parasite infections could be present (refugees, travelers form endemic areas, adopted children, new arrived migrants..).

This study was partially funded by Menarini, S.A., distributor of SediMAX2 in Spain. The funder had no role in the study design, data collection or analysis, the decision to publish, or the preparation of the manuscript.

## Data Availability

All data produced during this study are included in this published article.
